# The quest for meaning and self-reconstruction: a qualitative study on how sports participation enhances well-being in older adults

**DOI:** 10.3389/fpubh.2026.1883103

**Published:** 2026-06-30

**Authors:** Hezhi Liu

**Affiliations:** College of History and Culture, Chengdu Sport University, Chengdu, China

**Keywords:** older adults, pursuit of meaning, qualitative research, self-reconstruction, sports participation, well-being

## Abstract

**Background:**

Population aging has become a profound structural challenge in contemporary China. While extensive quantitative research has established a positive correlation between sports participation and older adults’ subjective well-being, the underlying mechanisms—how and why this enhancement occurs in everyday life—remain largely undertheorized.

**Purpose:**

This study aims to move beyond variable-centered explanations and explore the deep meanings and practical logic through which older adults construct well-being via sports participation. Specifically, it asks: (a) What multidimensional, rich experiences of well-being do older adults encounter? (b) How is this well-being actively constructed through sports participation as a “practice of meaning”? (c) How does this constructive process reshape older adults’ perceptions and narratives of self, aging, and social relationships?

**Methods:**

Adopting a constructivist grounded theory approach (1), the study conducted in-depth interviews and participatory observations with 24 older adults (aged 60–77) who had been engaged in organized sports activities (e.g., Tai Chi, square dancing, gateball, cycling) for at least 6 months. Data were analyzed using open, axial, and selective coding with NVivo 12.

**Results:**

Findings from all 24 participants reveal that sports participation serves as a proactive “practice of life subjectivity” during life transitions. The enhancement of well-being stems from a four-dimensional meaning-making process shared across participants: (a) Bodily return—from the “ailing body” to the “capable body,” regaining a sense of life control; (b) Socio-temporal reconstruction—rebuilding daily rhythms through regular activities and producing “routinized communitas” via embodied synchronous collective practices, thereby generating emotional energy on a regular basis; (c) Social role transformation—sports communities become new social stages for acquiring new roles and accumulating novel, low-pressure social capital; (d) Narrative identity shift—integrating sports experiences into life stories, re-authoring aging from a decline narrative into a progressive, value-driven narrative.

**Conclusion:**

A “meaning-centered” theoretical model is proposed, wherein the pursuit of meaning drives self-reconstruction, which in turn generates well-being. Sports participation is essentially a core arena for psychological empowerment and meaning-making. Policy and practice should shift from “providing services” to “enabling practices” to support older adults’ spontaneous meaning creation.

## 1 Introduction

Population aging represents a profound structural transformation in contemporary China. By the end of 2024, individuals aged 60 and above accounted for 22% of the total population (1), signaling that China has entered a deeply aged society. Against this backdrop, “active aging” has evolved from an international conceptual advocacy into a pressing governance issue. As scholars have noted, “developing senior sports is a crucial measure for implementing the national strategy of actively responding to population aging” (2). However, when I shift my gaze from macro-level policies to micro-level lived experiences, older adults face a series of concrete and profound life transitions. Retirement means the sudden loss of professional identity and the cessation of a regular social clock. The empty-nest phase dissolves core family roles and reduces everyday emotional interactions. Meanwhile, natural physical decline often accompanies weakened self-efficacy and the imposition of stigmatizing “aging” labels. Research indicates that over 50% of older adults in China live alone or in empty-nest situations, and nearly 30% experience varying degrees of depressive symptoms (3). These interrelated structural, psychological, and bodily challenges constitute real barriers that older adults must navigate in their pursuit of well-being.

Sports participation has been widely recognized as an effective means to address these challenges and enhance subjective well-being among older adults. Existing studies, largely based on large-scale survey data and structural equation modeling, have repeatedly verified that sports participation significantly improves well-being through mediating pathways such as health promotion (4), social networking (5), and psychological resilience (6). Other research from an active aging perspective has examined macro-level issues including senior sports policy (7), integrated physical-activity and care models (8), and smart sports services (9). Collectively, these findings have successfully mapped a variable-relationship diagram linking sports participation and well-being, confirming their positive association at a macro level.

Beyond quantitative evidence, a growing body of qualitative research has explored the lived experiences of sports and leisure participation across diverse populations. Studies have examined how physical activity fosters meaning-making among individuals facing life transitions, such as retirement, illness, or migration [e.g., (10–12)]. In the context of aging, narrative and phenomenological inquiries have revealed that sports participation can provide a sense of continuity, social integration, and personal growth (13, 14). However, most of these studies have focused on Western populations or specific clinical groups. The present study extends this line of inquiry by investigating a Chinese urban sample, thereby contributing cross-cultural insights into how meaning-making through sports operates in a non-Western, rapidly aging society.

However, when the research lens is overly focused on correlation coefficients and mediating effect sizes, a critical methodological issue emerges: how do these mediating pathways—derived from large-scale data—actually unfold in the concrete, everyday contexts of older adults’ lives? Quantitative paradigms operationalize “sports participation” as frequency and type, and “well-being” as scale scores. While this approach contributes to scientific objectivity, it methodologically brackets out deep explorations of the lived experiences and subjective agency behind the statistics (15). As qualitative studies have shown, even well-fitting models fail to reveal what concrete practices—such as square dancing or Tai Chi (16)—truly mean to older adults confronting the aforementioned real-life dilemmas. In other words, existing research, while establishing that sports participation is beneficial, remains largely under-explanatory regarding the micro-level mechanisms through which it helps older adults cope with order disruption, social disconnection, and crises of meaning—that is, the why and how questions.

This explanatory gap calls for a fundamental shift in research perspective: from empirical testing of variable relationships to an in-depth interpretation of older adults’ subjective worlds of meaning. From a hermeneutic phenomenological perspective, sports participation among older adults is far more than an isolated health intervention; it is more likely a “practice of meaning” (17)—an active effort to resist disorder, reconstruct daily rhythms, and pursue significance in response to ruptured life order. It is about identity reconstruction and value confirmation; not just an individual behavior but an agentic process of reconnecting with society. This perspective shift aligns with recent calls within sport studies to revisit the methodological value of qualitative research (18) and to adopt an approach that “returns to the things themselves.”

To address this gap, I adopt such a perspective shift: moving from empirical testing of variable relationships to an in-depth interpretation and theoretical generation of how sports participation shapes older adults’ well-being experiences. Adopting an integrative framework combining hermeneutic phenomenology and constructivist grounded theory, this study situates sports participation within older adults’ everyday life flows and unique life histories. This study addresses the following research questions:

(1) What are the multidimensional, rich connotations and subjective experiences of well-being that older adults encounter through sports participation?
(2) How is this unique well-being experience actively constructed and generated through sports participation as a “practice of meaning”?
(3) How does this constructive process influence and reshape older adults’ perceptions and narratives of self, aging, and social relationships?

By investigating these questions, this study aims to move beyond linear explanatory models and reveal the intrinsic, vivid, and meaning-laden complex mechanisms through which senior sports participation enhances well-being. In doing so, it seeks to provide embodied, life-world-grounded theoretical support and practical insights for optimizing China’s senior sports support system and advancing the “Active Aging” and “Healthy China” strategies.

## 2 Theoretical framework

This study is guided by an integrative theoretical framework that draws on three intersecting intellectual traditions: hermeneutic phenomenology, constructivist grounded theory, and a set of sensitizing concepts from interaction ritual chain theory and narrative identity theory. Together, these perspectives enable us to move beyond variable-centered explanations and toward a processual, meaning-centered understanding of how sports participation enhances well-being in later life.

### 2.1 Hermeneutic phenomenology as philosophical grounding

At the most fundamental level, this study adopts a hermeneutic phenomenological orientation (19). Phenomenology directs my attention to the “lived experience” (Lebenswelt) of older adults as they engage in sports activities. It asks: What is it like to be an older person participating in Tai Chi, square dancing, or cycling? This philosophical commitment ensures that my inquiry remains anchored in the concrete, embodied, and affective dimensions of well-being, rather than abstracting it into decontextualized variables. Hermeneutics adds an interpretive layer: I recognize that experiences of well-being are not simply “given” but are actively mediated through language, narrative, and social meaning-making. Thus, this study’s goal is not to produce objective measurements but to offer a rich, interpretive understanding of how older adults themselves experience, articulate, and imbue with meaning their sports participation.

### 2.2 Constructivist grounded theory as methodological logic

To systematically generate a substantive theory from the ground up, this study adopts constructivist grounded theory (20). Unlike more positivist versions of grounded theory [e.g., (21)], the constructivist approach acknowledges that knowledge is co-constructed between researchers and participants, and that the resulting theory is an interpretative rendering of the studied world rather than a passive discovery of an objective reality. This epistemological stance aligns closely with this study’s research questions, which focus on how older adults actively construct well-being through meaning-making practices.

Key features of this approach include: (a) simultaneous data collection and analysis; (b) pursuing emerging themes through theoretical sampling; (c) constructing middle-range categories rather than grand theories; and (d) prioritizing participants’ voices and agency. By following systematic coding procedures (open, axial, selective) while remaining flexible and reflexive, this study aims to produce a grounded theory that is faithful to the empirical material and useful for understanding the processes of self-reconstruction in later life.

### 2.3 Sensitizing concepts: interaction ritual chains and narrative identity

While grounded theory encourages researchers to remain open to emergent themes, it also acknowledges the value of “sensitizing concepts”—theoretical ideas that provide a loose orientation without predetermining findings. Two such concepts inform my analysis.

First, interaction ritual chain theory (22) argues that when people assemble in physical copresence, share a common focus of attention, and synchronize their actions and emotions, they can produce collective effervescence, emotional energy, and feelings of group solidarity. This theory has traditionally been applied to episodic, bounded rituals (e.g., religious ceremonies, public assemblies). This study extends its logic to ask: Can routine, low-intensity, but highly regular collective sports activities (e.g., daily square dancing, weekly gateball games) generate similar affective states? And if so, what might these “routinized rituals” mean for older adults facing social isolation and emotional loneliness?

Second, narrative identity theory (23) proposes that individuals construct a sense of self by weaving disparate life events into a coherent, meaningful story. This story evolves over time and is dialogically shaped by cultural narratives and social relationships. In later life, when career-driven narratives lose their force, a “narrative vacuum” may emerge. This study uses this concept to explore whether sports participation provides new plot elements (e.g., challenges, achievements, recognitions) that enable older adults to re-author their identities from decline-oriented to growth-oriented narratives.

Together, these three theoretical pillars—phenomenological grounding, constructivist grounded methods, and sensitizing concepts from ritual and narrative theories—provide a robust yet flexible framework for investigating the quest for meaning and self-reconstruction through sports participation in older adulthood.

### 2.4 Well-being theories as conceptual background

Because this study centers on older adults’ well-being, it is necessary to briefly situate the inquiry within the major theoretical traditions of well-being. Three influential approaches exist. The “hedonic” perspective defines well-being as the experience of pleasure and avoidance of pain (24). The “eudaimonic” perspective emphasizes self-realization, living in accordance with one’s true self, and the pursuit of meaning and virtue (25). The “desire-satisfaction” theory holds that well-being consists in the fulfillment of an individual’s informed desires, whatever those desires may be (26).

While the findings resonate with elements of eudaimonic well-being—particularly the emphasis on meaning and purpose—the desire-satisfaction framework offers a particularly productive dialogue with the concept of self-reconstruction developed in this study. Desire-satisfaction theory does not prescribe a fixed set of human goods; rather, it focuses on whether individuals are able to achieve what they genuinely want. In the context of later life, where previous desires (e.g., career advancement, intensive child-rearing) may have become obsolete or unattainable, older adults develop new desires—for bodily agency, social belonging, valued roles, and a positive narrative identity. Sports participation enables the satisfaction of these newly formed desires, thereby generating well-being. This theoretical alignment strengthens the philosophical grounding of the study and clarifies why self-reconstruction—as a process of reorienting one’s desires toward achievable and meaningful goals—is central to well-being enhancement in later life.

## 3 Methods

### 3.1 Research paradigm

This qualitative study is situated within the interpretivist–constructivist paradigm. This study assumes that reality is socially constructed, multiple, and context-dependent, and that the goal of research is to understand the meanings that people attach to their experiences. Given that this study’s research questions seek to explore the how and why of well-being construction—a process-oriented, meaning-centered phenomenon—a qualitative paradigm is not only appropriate but essential. Within qualitative traditions, constructivist grounded theory (20) was selected as the core methodological logic, supplemented by hermeneutic phenomenological sensitivity (19) to ensure immersion in lived experience.

### 3.2 Participants and sampling

Participants were recruited using a combination of purposive sampling (initial stage) and theoretical sampling (subsequent stages). Inclusion criteria were designed to ensure rich, relevant data:

1. Age ≥ 60 years. This criterion aligns with the official definition of older adults in China and targets the life phase characterized by retirement, role transitions, and bodily changes.
2. Continuous participation in organized sports activities for ≥ 6 months. The “continuous participation” threshold ensures that participants have moved beyond initial trial stages and can narrate stable, routinized experiences. The “organized activities” criterion (e.g., community square dance teams, Tai Chi coaching stations, gateball associations, cycling clubs) was a theoretically driven decision: such settings naturally contain rules, roles, regular interactions, and shared goals, maximizing the likelihood that core social-psychological processes (e.g., social bonding, role acquisition, and belonging) would emerge. Purely individual, unorganized activities (e.g., solitary walking) were excluded because they offer weaker social interaction dimensions.

To capture a wide range of experiences, initial sampling sought maximum variation in gender, age subgroup (young-old vs. old-old), sport type, living arrangement (alone vs. with others), and educational background.

Participants were recruited from three urban districts in Chengdu, Sichuan Province, China. Recruitment channels included community activity centers (*n* = 12), local sports associations (e.g., gateball associations, square dance teams; *n* = 8), and snowball sampling through initial participants (*n* = 4). The author visited community centers and sports venues in person, explained the study purpose, and invited eligible individuals to participate. All recruitment materials were approved by the institutional review board.

A total of 24 older adults participated (see Table 1). Data collection and analysis proceeded iteratively until theoretical saturation was reached: after analyzing the 22nd participant’s data, the core category of “self-reconstruction” was fully developed with no new theoretical insights; two additional participants were interviewed to confirm saturation.

**Table 1:** Demographic information of participants (*N* = 24)phic.

ID	Pseudonym	Gender	Age	Primary sport	Years participating	Former occupation	Living arrangement
P01	Ms. Liu	F	68	Tai Chi	5	Primary teacher	With spouse
P02	Mr. Zhang	M	72	Brisk walking	3	Factory technician	Alone
P03	Ms. Li	F	70	Square dance	4	Accountant	With spouse
P04	Aunt Wang	F	65	Square dance	2	Salesperson	With adult child
P05	Uncle Chen	M	74	Gateball	6	Cadre	With spouse
P06	Grandma Yang	F	69	Tai Chi	3	Nurse	Alone
P07	Mr. Wang	M	65	Drum corps	2	Driver	With spouse
P08	Grandpa Liu	M	73	Brisk walking	4	Farmer	With spouse
P09	Aunt Zhao	F	66	Square dance	3	Textile worker	With adult child
P10	Uncle Sun	M	71	Gateball	5	Technician	Alone
P11	Grandma Zhou	F	76	Tai Chi	7	Teacher	With spouse
P12	Ms. Chen	F	67	Waist drum	3	Accountant	With spouse
P13	Mr. Zhao	M	71	Gateball	4	Driver	With spouse
P14	Uncle Li	M	69	Cycling	2	Engineer	With spouse
P15	Uncle Sun	M	70	Gateball	3	Worker	With adult child
P16	Aunt Wu	F	68	Square dance	4	Waitress	Alone
P17	Grandpa Zheng	M	77	Tai Chi	8	Doctor	With spouse
P18	Grandma Lin	F	72	Brisk walking	2	Teacher	Alone
P19	Uncle Guo	M	63	Cycling	1	Civil servant	With spouse
P20	Mr. Sun	M	75	Winter swimming	6	Engineer	With spouse
P21	Aunt Huang	F	64	Square dance	2	Accountant	With spouse
P22	Mr. Ma	M	70	Tai Chi	5	Worker	Alone
P23	Grandma Song	F	73	Gateball	4	Teacher	With adult child
P24	Ms. Gao	F	66	Cycling	4	Civil servant	With spouse

### 3.3 Data collection

Data were collected through two primary methods: in-depth semi-structured interviews and participatory observation, supplemented by field notes and researcher memos.

#### 3.3.1 Interviews

Each participant was interviewed individually, with interviews lasting 60–90 min. Interviews were conducted in quiet, familiar settings (e.g., community activity rooms, participants’ homes) after obtaining informed consent. All interviews were audio-recorded and transcribed verbatim. The semi-structured interview guide covered four thematic areas: (a) life history before and after retirement (transitions, challenges, identity shifts); (b) trajectory of sports participation (initiation, continuation, occasional interruptions); (c) embodied and emotional experiences during activities (physical sensations, affective fluctuations, social interactions); and (d) personal meanings attributed to sports participation (role in current life, value, significance for aging).

All interviews were conducted in Mandarin Chinese, the native language of both the participants and the author. Audio recordings were transcribed verbatim in Chinese. For the purpose of this English-language manuscript, direct quotations were translated into English by the author, who is bilingual. To ensure accuracy and preserve the original meaning, a back-translation procedure was employed: a second bilingual researcher (not involved in the study) independently translated a random sample of 20% of the quotations from English back into Chinese, and the two Chinese versions were compared. No significant discrepancies were found. All participant codes and contextual information were retained throughout the translation process.

#### 3.3.2 Participatory observation

To triangulate interview data and gain contextualized, holistic understanding, the author conducted participatory observation in natural settings where older adults engage in sports: urban parks, public squares, community sports centers. Observations focused on the spontaneous unfolding of activities, interaction patterns among participants, body language, group atmosphere, and use of spatial environment. The researcher adopted a “participant-as-observer” role, occasionally engaging in informal conversations to deepen immersion.

### 3.4 Data analysis

Theoretical saturation was defined as the point at which new data no longer generated new properties or dimensions of the core category “self-reconstruction” and all emergent themes were well-developed [(20), p. 113]. The process involved three coding stages:

#### 3.4.1 Open coding

This study performed line-by-line coding, breaking down the transcribed data into discrete incidents and assigning them initial conceptual labels. Codes were derived from participants’ own language as much as possible (e.g., *“feeling that the body is mine again,” “found my tribe”*). This process generated 86 initial concepts from the full interview corpus.

#### 3.4.2 Axial coding

This study then compared and grouped the open codes into higher-order categories, identifying relationships between categories. For example, codes such as “making new friends,” “helping each other with errands,” and “having tea and meals together” were grouped into the category “constructing new social networks.” Through constant comparative analysis, 12 core categories were synthesized.

#### 3.4.3 Selective coding

**Figure 1:**
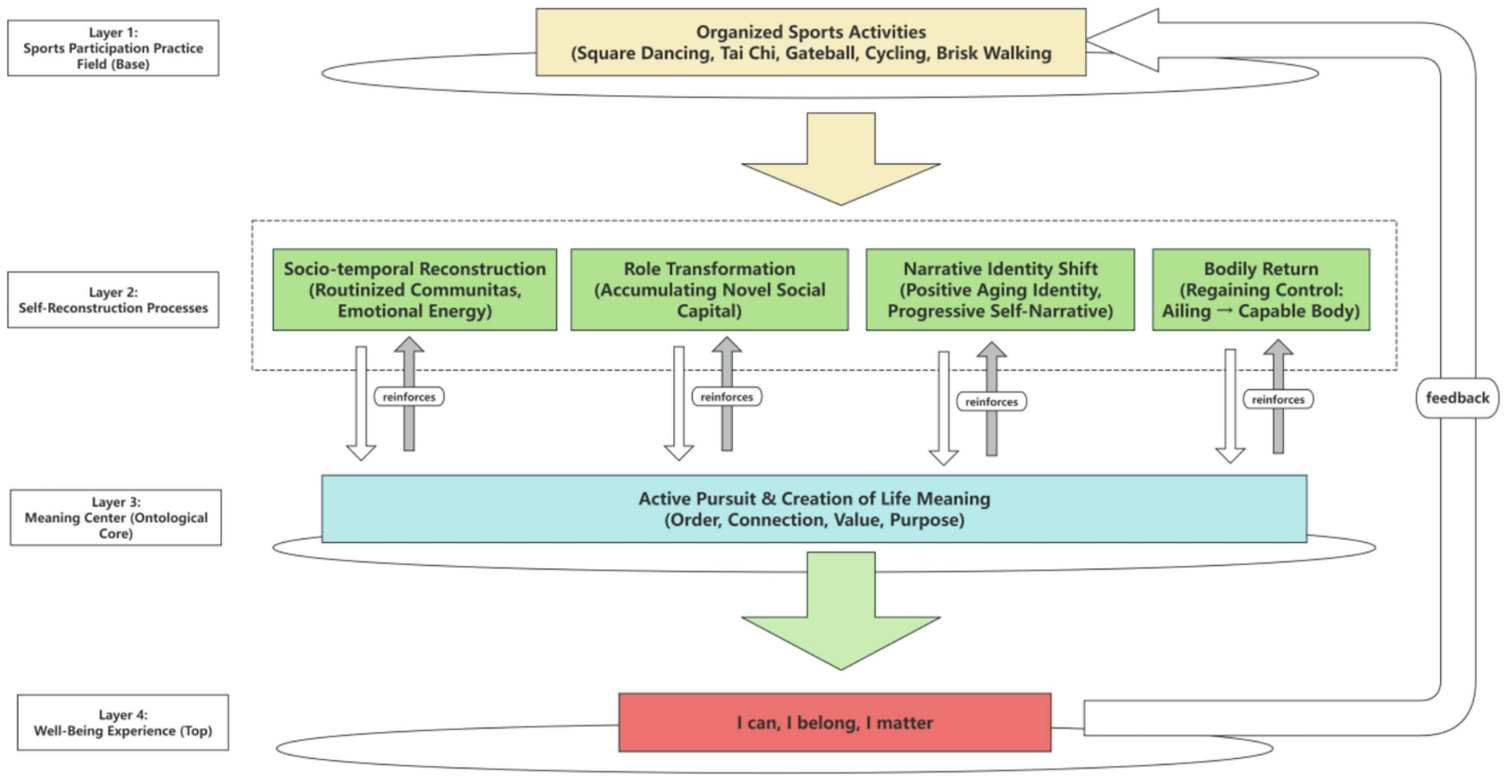
The “Meaning-Centered” model of sports participation enhancing well-being in older adults.

Finally, this study systematically analyzed all categories to identify a core category that could unify the others. The core category that emerged was *“self-reconstruction.”* All other categories—including *“regaining bodily control,” “reconstructing daily rhythms,” “creating routinized communitas,” “role transformation,”* and *“narrative identity shift”*—were integrated into a storyline centered on self-reconstruction. Based on this integration, the theoretical model was constructed (see Figure 1 in the Discussion section).

All coding was performed by the author, who is the sole researcher. To manage interpretive subjectivity, several strategies were employed. First, a coding audit trail was maintained, documenting all decisions about code creation, merging, and category formation. Second, regular peer debriefing sessions were held with two qualitative researchers from other disciplines (one in public health, one in sociology) who were not involved in the study; they reviewed 30% of the coded transcripts and provided feedback on the consistency and plausibility of the emerging categories. Disagreements were resolved through discussion, and the final coding structure was adjusted accordingly. Third, a reflective journal (see Section 3.5) was used to bracket pre-existing assumptions about aging and sports participation Table 2.

**Table 2:** Example of the coding processe.

Raw data excerpt (translated)	Open coding	Axial category	Selective core category
“Right after retirement, I had aches and pains everywhere. Now I feel this body is mine again, it listens to me.” (P01)	Bodily anxiety, bodily ownership, bodily control	Regaining bodily control	Self-reconstruction
“Rain or shine, 7:30 p.m. sharp. This is like a happy ‘shift’.” (P03)	Regularity, ritual, psychological dependence, time structuring	Reconstructing daily rhythms	Self-reconstruction
“We strike on the same beat, breathe together. I feel that I’m part of this big family.” (P07)	Synchronized action, collective excitement, boundary dissolution, emotional fusion	Creating routinized communitas	Self-reconstruction
“Here everyone teases me, says I’m the one who brings the joy. I feel useful.” (P13)	Acquiring new role, being needed by the group, value reaffirmation	Social role transformation	Self-reconstruction
“Each time I get into the water, I conquer myself. I want to live a life that even young people admire!” (P20)	Self-challenge, resisting aging stereotypes, life passion	Constructing positive aging identity	Self-reconstruction
“The sisters I dance with aren’t like old colleagues with all those grudges. It’s just playing together. If someone has a problem, everyone is eager to help.” (P01)	Egalitarian interaction, instrumental support, emotional support	Accumulating novel social capital	Self-reconstruction

### 3.5 Trustworthiness and ethical considerations

To ensure the rigor of this qualitative study, this study employed multiple strategies recommended for constructivist grounded theory (20, 27).

#### 3.5.1 Triangulation

This study collected data from multiple sources (interviews, observations, field notes, memos) and cross-checked findings across participants and methods.

#### 3.5.2 Member checking

Preliminary findings and interpretations were returned to a subset of participants for feedback. All participants confirmed that the themes resonated with their experiences.

#### 3.5.3 Peer debriefing

Regular meetings with a research group not involved in data collection were held to discuss emerging themes, challenge interpretations, and reduce researcher bias.

#### 3.5.4 Thick description

Rich, detailed accounts with extensive participant quotes are provided to enable readers to assess the transferability of findings to their own contexts.

#### 3.5.5 Researcher reflexivity

The author, a male researcher in his 50s, maintained a reflective journal throughout the study, documenting assumptions, emotional responses, and how his positionality (younger, not yet retired, initially an “outsider” to the aging experience) might shape data collection and interpretation. Prolonged engagement (over six months in the field) helped build trust and reduce power imbalances.

#### 3.5.6 Ethical considerations

This study received approval from the institutional review board of the authors’ affiliated university. All participants were fully informed about the purpose of the study, their rights to withdraw at any time, and the measures taken to protect confidentiality. Written informed consent was obtained. All names and identifying details have been anonymized.

## 4 Results

The results of this study demonstrate how older adults construct well-being through sports participation as a multidimensional process of self-reconstruction. Through systematic comparative analysis of interview transcripts, observational field notes, and researcher memos, four interrelated and progressively deepening themes emerged. These themes collectively illustrate the complete practical trajectory through which older adults, by engaging in organized sports activities, achieve self-reconstruction across bodily, socio-temporal, relational, and narrative dimensions.

### 4.1 Theme 1: the return of the body and the reconstruction of a sense of control

All 24 participants described this theme. For many of them, the initial motivation for sports participation originated from a profound anxiety about losing bodily control. As aging and retirement brought physical decline, the body shifted from a familiar companion to a source of pain, constraint, and loss of agency. One participant described this experience:

*“Right after retirement, I always had aches and pains here and there, constantly wondering if something new was wrong with me… I felt like I had become a regular at the hospital.”* (P01, female, 68, Tai Chi).

This anxiety is not merely individual worry; research consistently shows that self-rated health and physical functioning are core predictors of subjective well-being among older adults (3, 28). However, the data reveal the embodied mechanism underlying this statistical association.

Sports activities became a key practice through which participants re-engaged with their bodies and regained mastery. The core transformation was from being controlled by the body to controlling the body. P01 continued:

*“Later I started practicing Tai Chi. At first I could not even connect the “cloud hands” movement smoothly; I was out of breath. But now I feel this body is mine again, it listens to me. If I do not practice one day, I feel stiff, as if I owe it something.”*

This quote illustrates how sports participation transforms the body from a passive symbol of illness into an active, dialogical, and manageable life tool.

This sense of control was further reinforced through the continuous conquest of small, concrete challenges, which translated into tangible self-efficacy. Mr. Zhang (P02, male, 72, brisk walking), a retired factory technician, proudly shared his achievement map:

*“I used to get winded after two laps in the park. Now I can do five laps! My son bought me a pedometer, and every day I watch the numbers go up—what a great feeling! Last month I joined the community’s “10,000 Steps Challenge” and I completed it. This means I’m still capable. I’m not over the hill yet.”*

Similarly, Uncle Sun (P15, male, 70, gateball) found confirmation in skill improvement:

*“At the beginning I could barely hit the ball. Now I can not only hit it but also think about strategy—making “double-touch shots.” My mind and my eyesight have not rusted!”*

These *“I can do it”* embodied experiences significantly counteracted the feelings of helplessness and social marginalization associated with aging. Sports participation transformed abstract health concerns and aging anxieties into daily perceivable, measurable, and conquerable small victories, thereby enabling older adults to reaffirm their agency over life. This bodily return constitutes the most fundamental foundation of their well-being experience.

### 4.2 Theme 2: the reconstruction of daily rhythms and the emotional energy of “routinized communitas”

Retirement brings not only the loss of professional identity but also the sudden disruption of the social time clock, leaving large, unstructured voids of time accompanied by feelings of loss, confusion, and a sense of worthlessness. Sports activities, with their inherent regularity and ritualistic qualities, successfully filled these voids with new social structure and emotional meaning, becoming powerful tools to combat life disorder.

Observations revealed that older adults’ sports activities followed remarkably consistent temporal and spatial patterns, constituting a stable social clock within urban environments. Every morning and evening, parks and squares would reliably gather exercising groups with precise schedules and fixed territories. Ms. Li (P03, female, 70, square dance) described this with a sense of ritual:

*“Rain or shine, 7:30 p.m. sharp—when the music starts and the old sisters take their positions, it’s like… like punching in for work, but this is a happy “shift.” If we stop because of wind or rain, I feel off the whole day, my heart feels empty, as if the day has not been fully lived.”*

This highly structured participation not only filled time but also endowed it with purpose and anticipation, transforming meaningless *“killing time”* into productive *“investing time.”*

More importantly, during these highly synchronous collective activities, this study observed an intense yet temporary state of emotional fusion. When individuals moved their bodies in a shared rhythm, breathed together, and focused their attention collectively, everyday social hierarchies and identity differences seemed to temporarily dissolve, replaced by a strong sense of collective consciousness and emotional effervescence. Mr. Wang (P07, male, 65, drum corps) excitedly recalled a large public performance:

*“So many of us, striking on the same beat, breathing together—that feeling, hey! I was totally absorbed… I felt that I was part of this big family. Strong! My whole body was filled with warmth!”*

This form of collective emotional experience—emergent from my empirical data—shares profound family resemblances with anthropologist Victor Turner’s concept of “communitas” (29), which refers to an anti-structural, egalitarian, and affective state of togetherness that transcends everyday social differentiations. However, the experience created by Chinese urban older adults through daily or weekly routine sports activities differs from Turner’s episodic, sacred-ritual-based communitas in two important ways. First, routinization: it does not occur in spaced-out, special rituals but is embedded in the fabric of everyday life, becoming an expected and repeatable “emotional refueling station.” Second, sustainability: this experience is maintained and reinforced through high-frequency repetition, rather than being a one-time liminal episode. The data thus suggest the existence of a routinized communitas—a mundane yet powerful form of collective effervescence that regularly produces emotional energy for participants, helping them counter the social atomization and loneliness that often accompany aging.

### 4.3 Theme 3: the transformation of social roles and the accumulation of valued social capital

Retirement often means the abrupt loss of a clear social role and the sharp shrinkage of social networks, leading to role disruption and relational poverty. Sports communities, the data show, function as substitute social stages and identity laboratories. Based on shared interests, personal qualities, and present contributions—rather than past social status—older adults acquire new roles, respect, and support, thereby accumulating a new form of valuable social capital.

Many participants experienced a creative regeneration of social identity within their sports communities. Ms. Chen (P12, female, 67, waist drum), formerly a factory accountant, was now respectfully called “Captain Chen” in her waist drum team due to her organizational skills and fair-mindedness. She explained:

*“At my job I just followed routines. Now I manage dozens of people in rehearsals and performances, and everyone follows my arrangements. I feel even busier and more useful than before retirement.”*

Mr. Zhao (P13, male, 71, gateball), previously a driver, became the “class clown” and “chief logistics officer” of his gateball team because of his humor and warm-heartedness. He said with a smile:

*“At work I was just a driver; supervisors could barely remember my name. Here, everyone teases me, says I’m the one who brings the joy. If someone forgets their water bottle or breaks a mallet, they come to me first. I feel useful, like an indispensable “old ox” in this group.”*

These new roles were not imposed from above but emerged organically from participation, providing participants with recognition, agency, and a sense of mattering.

The weak-tie friendships built on shared interests and egalitarian participation constituted a unique and flexible social support network. This network provided both instrumental support (e.g., daily errands, information sharing) and, crucially, emotional support (e.g., listening, encouragement, affirmation). Ms. Liu (P01) elaborated:

*“The sisters I dance with aren’t like old colleagues with all those grudges and hierarchies. It’s just playing together, pure. If someone has a problem—say, needing someone to pick up groceries, book a hospital appointment, or accompany them to the doctor—we post it in the group chat, and everyone is eager to help. This kind of connection is sometimes more solid than relatives.”*

This low-pressure, highly flexible “social buffer zone”—constructed outside the immediate family circle—greatly enhanced participants’ sense of social security, belonging, and integration. It represents a key indicator of social health and psychological well-being in later life.

### 4.4 Theme 4: the shift in life narratives and the construction of a positive aging identity

Beyond being a daily activity, sports participation was actively integrated by many participants into their life stories, becoming a central narrative tool for coping with aging and reconstructing self-identity. Through this practice, they successfully overturned the dominant cultural narrative of aging-as-decline and replaced it with an agentic, progressive narrative of growth, challenge, and value regeneration.

The story of Mr. Sun (P20, male, 75, winter swimming) exemplifies this narrative transformation. He positioned himself as a “life challenger” and “age rebel”:

*“Other people think that at my age, winter swimming is torture. I see it as conquest. Every time I get into the water, I conquer myself—conquering the cold, and also conquering that inner voice that says “you are old, you cannot do this.” Am I not a human being just because I’m old? Cannot I have passion? I want to live a life that even young people admire! My bones and muscles are stronger than many office-sitting young people.”*

In his narrative, age and body were no longer markers of decline but medals showcasing courage, perseverance, and life vitality.

For many participants, sports participation helped construct a positive aging identity. They no longer saw themselves as passive “old people” or burdens, but as active “happy-aging folks” or “senior youth.” Ms. Gao (P24, female, 66, cycling) offered a representative summary:

*“I feel that my life really began after retirement… They call me “sunset red”? I call it my “second spring”—life starting anew!”*

The analysis reveals that participants systematically wove the challenges, achievements, and social rewards from sports participation into their retrospective and prospective life narratives, thereby creating a progressive self-narrative. In this narrative, aging is no longer a pure decline story but is re-signified as an ongoing chapter of exploration, learning, mastery, and value regeneration.

The finding extends narrative identity theory (23). While McAdams emphasizes that individuals construct identity by integrating life experiences into a coherent story, the theory has traditionally focused on life-span grand narratives or clinical therapeutic settings. This study concretely shows how, during the life transition of later life in contemporary China, sports participation serves as a narrative engine and story-material repository. Facing a narrative vacuum after retirement, participants actively used the quantifiable achievements (step counts, lap numbers), immediate social feedback (group recognition, role conferral), and positive bodily perceptions provided by sports activities to counter the culturally dominant decline narrative of aging. They authored a “second-life” script emphasizing agency, competence, and future orientation. Thus, these findings extend the theory by revealing a practice-generated pathway to narrative identity—achieved through embodied, group-based, daily practices rather than through introspective life review alone.

## 5 Discussion

This study aimed to move beyond variable-centered, linear explanations of the relationship between sports participation and well-being among older adults. Through an in-depth qualitative investigation grounded in constructivist grounded theory and hermeneutic phenomenology, this study have shown that sports participation enhances well-being not through simple cause-and-effect pathways but through a multidimensional, dynamic process of self-reconstruction. Below, this study integrates the four emergent themes, present a theoretical model, articulate three key theoretical contributions, and discuss limitations and future directions.

### 5.1 Addressing the research questions

The findings directly answer the three research questions posed in the introduction.

(a) What multidimensional well-being experiences do older adults encounter through sports participation? Participants reported well-being as comprising four interwoven dimensions: (i) a sense of bodily control and mastery (Theme 1); (ii) the emotional energy derived from routinized collective synchrony (Theme 2); (iii) the recognition and value obtained from new social roles (Theme 3); and (iv) a progressive, growth-oriented life narrative (Theme 4). These dimensions extend beyond hedonic pleasure to include eudaimonic and desire-satisfaction components, as discussed in Section 2.4.
(b) How is well-being actively constructed through sports participation as a “practice of meaning”? Well-being is not passively received but actively built through four meaning-making mechanisms: bodily return (transforming the ailing body into a capable agent), socio-temporal reconstruction (creating routinized communitas that regularly produces emotional energy), social role transformation (accumulating flexible social capital), and narrative identity shift (re-authoring aging as a progressive story). Each mechanism involves the pursuit and creation of meaning—order, connection, value, and purpose. These mechanisms do not operate in isolation; they form a cascading logic where bodily control enables temporal structuring, which facilitates social connection, which in turn supplies narrative materials for identity reconstruction.
(c) How does this process reshape older adults’ perceptions of self, aging, and social relationships? Through sustained participation, participants came to see themselves not as passive recipients of decline but as active agents capable of growth. Aging was re-signified from a period of loss to one of “second spring” or continued development. Socially, participants built new, low-pressure networks outside family circles, experiencing themselves as valued contributors rather than burdens. These findings resonate strongly with the desire-satisfaction theory of well-being (26), which holds that well-being consists in the fulfillment of an individual’s informed desires. As will be elaborated in Section 5.3.5, each of the four emergent themes can be understood as the satisfaction of newly formed desires that emerge in later life—desires for bodily agency, temporal order, social belonging, valued roles, and a coherent life narrative.

### 5.2 The “meaning-centered” theoretical model

Based on the integrative analysis, this study proposes a “meaning-centered” theoretical model of how sports participation enhances well-being in older adults (see Figure 1). The model consists of four hierarchical layers:

#### 5.2.1 Layer 1 (Base): sports participation practice field

Organized sports activities (square dancing, Tai Chi, gateball, cycling, brisk walking, etc.) provide the embodied, interactive arena where meaning-making begins.

#### 5.2.2 Layer 2: self-reconstruction processes

Sports participation triggers four concurrent processes—bodily return (regaining control), routinized communitas (emotional energy production), role transformation (social capital accumulation), and narrative shift (positive aging identity).

#### 5.2.3 Layer 3: meaning center

These four processes are not independent “mediators” but are unified by a common ontological core: the active pursuit and creation of life meaning. The meaning center both drives and is reinforced by self-reconstruction. From a desire-satisfaction perspective, the meaning center represents the ongoing process of forming, pursuing, and fulfilling informed desires for bodily agency, social belonging, valued roles, and narrative coherence.

#### 5.2.4 Layer 4: well-being experience

Through the recursive cycle of pursuing meaning and enacting self-reconstruction (each reinforcing the other), older adults achieve a deep-seated sense of well-being summarized as “I can, I belong, I matter.”

The arrows in Figure 1 represent the following relationships:

- Solid upward arrows from Layer 1 to Layer 2 indicate that sports participation enables the four self-reconstruction processes. For example, the regularity and physical demands of Tai Chi or square dancing directly produce bodily control and temporal structure.
- Solid upward arrows from Layer 2 to Layer 3 indicate that each self-reconstruction process contributes to the meaning center. Bodily control provides a sense of agency; routinized communitas supplies emotional energy; role transformation offers social value; narrative shift gives coherence.
- Solid upward arrow from Layer 3 to Layer 4 signifies that realized meaning directly generates well-being (the “I can, I belong, I matter” state).
- Dashed downward arrow from Layer 4 back to Layer 1 represents a feedback loop: the experience of well-being motivates continued sports participation, reinforcing the entire cycle.
- Dashed downward arrows from Layer 3 to Layer 2 represent recursive reinforcement: as meaning is confirmed, it strengthens each self-reconstruction process (e.g., feeling more purposeful encourages further bodily engagement). These relationships emerged inductively from participants’ narratives, particularly their statements about feeling “useful” (P13) or experiencing a “second spring” (P24), which led them to persist and even intensify their participation.

### 5.3 Theoretical contributions

This study’s findings extend and refine existing theoretical frameworks in three significant ways.

#### 5.3.1 From interaction ritual to routinized communitas: the everyday production of collective emotional meaning

Collins’ (22) interaction ritual chain theory powerfully explains how focused attention, bodily synchronization, and shared emotion can generate collective effervescence and emotional energy. However, Collins’ analysis primarily focused on episodic, bounded, and often high-intensity rituals (e.g., religious services, political rallies). This study reveals that among older adults engaged in daily or weekly sports activities, a routinized form of communitas exists. Unlike Turner’s (29) liminal communitas, which is transient and anti-structural, routinized communitas is embedded in the mundane, repeatable, and low-intensity fabric of everyday life. Yet it produces sustained emotional energy that serves as a buffer against social isolation and chronic loneliness.

This finding extends interaction ritual theory in two ways. First, it identifies a daily ritual mechanism that does not require extraordinary settings or high emotional arousal. Second, it demonstrates that even low-intensity, high-frequency rituals can generate meaningful solidarity and emotional resources, especially for populations facing social withdrawal. For older adults, the very predictability and regularity of square dancing or Tai Chi sessions provide a reassuring structure that transforms “empty time” into “emotionally invested time.”

#### 5.3.2 From psychological trait to narrative dynamics: re-theorizing the mediating mechanism

Existing quantitative studies often treat “psychological resilience” as a static mediating variable or individual trait that explains how sports participation improves well-being (6, 30). While useful, this conceptualization risks reifying resilience as something one has rather than something one does. The data suggest a complementary, process-oriented alternative: well-being enhancement is driven by a dynamic narrative meaning-making process. Older adults actively acquire new experiences, roles, and relationships through sports participation, and then weave these elements into a progressive life story.

This finding dialogues with McAdams’ (23) narrative identity theory. McAdams argued that individuals construct identity by integrating life experiences into a coherent, purpose-driven narrative. However, his theory has been predominantly applied to life review in clinical or retrospective contexts. This study extends it by showing how, during the post-retirement “narrative vacuum,” sports participation serves as a narrative engine—providing fresh plot elements (challenges, achievements, recognitions) that enable the authoring of a “second-life” script. Importantly, this narrative shift from decline to growth is not merely cognitive; it is embodied and socially enacted through daily practice. Thus, this study suggests that future research on sports and well-being should incorporate narrative dynamics alongside trait-based resilience measures.

#### 5.3.3 From functional maintenance to meaning creation: extending active aging frameworks

The World Health Organization’s active aging framework and its scholarly elaborations (e.g., (7, 8)) emphasize maintaining physical function, cognitive health, and social participation as keys to late-life quality. These findings strongly support the value of social participation, but they also reveal a dimension that active aging frameworks have insufficiently theorized: the subjectivity of older adults as meaning creators.

The study’s participants did not engage in sports merely to maintain health or stay busy. Their deeper motivation—and the actual outcome of their practice—was the pursuit and reconstruction of fundamental life meanings: order, connection, value, and purpose. They were not passive recipients of policy interventions but active meaning entrepreneurs of their own later lives. As one participant put it, *“They call me ‘sunset red’? I call it my ‘second spring’!”* This redefinition of life stage exemplifies the agentic dimension that active aging discourses often presuppose but rarely unpack.

Thus, the meaning-centered model complements and deepens active aging frameworks. Truly quality later life is not only about “aging in place” or “productive aging”—it is also about “meaningful aging.” Older adults in sports fields are not merely keeping fit; they are conducting a practical project of meaning-making through bodily mastery, temporal reconstruction, role transformation, and narrative re-authoring. This insight suggests that policy and practice should shift from a deficit-oriented “service provision” model to an asset-oriented “enabling practice” model—a point this study develops further in the conclusion.

#### 5.3.4 Distinguishing the meaning-centered model from related concepts

The present model shares conceptual ground with established frameworks of leisure, social participation, and successful aging, yet it offers distinct emphases. Leisure studies have long recognized that recreational activities can facilitate identity work and meaning-making (31, 32). However, leisure is often conceptualized as freely chosen, intrinsically motivated activity occurring in non-obligatory time. In contrast, the organized sports participation examined here—while voluntary—often carries a sense of commitment, regularity, and social obligation (e.g., “happy shift” as described by P03). This blend of obligation and enjoyment is characteristic of serious leisure (33) but has received less attention in aging research.

Similarly, social participation—a key pillar of active aging—encompasses any engagement in social, cultural, or civic activities. The meaning-centered model extends this by specifying *how* social participation generates well-being: through four interconnected mechanisms (bodily, temporal, relational, and narrative). Not all social participation automatically yields meaning; the present findings suggest that the presence of embodied synchrony (routinized communitas) and narrative integration are critical conditions. Thus, the model provides a processual explanation that complements the broader frameworks of active aging (34) and successful aging (42), which tend to focus on what older adults do rather than how and why it matters to them.

#### 5.3.5 Integrating desire-satisfaction theory with the empirical findings

The desire-satisfaction theory of well-being (26) offers a particularly productive lens for interpreting the four themes presented in this study. Unlike hedonic or eudaimonic frameworks that prescribe what “should”make people happy, desire-satisfaction theory focuses on whether individuals are able to achieve what they genuinely”want.” In later life, older adults’ desires often undergo a fundamental shift. Career advancement and intensive child-rearing may no longer be relevant or attainable. New desires emerge: to feel in control of one’s body, to fill empty time with purpose, to be needed and valued by others, and to see one’s life as a coherent, meaningful story. Each of the four themes directly corresponds to the satisfaction of such desires.

Theme 1 (Bodily return) addresses the desire for bodily agency. Participants expressed a strong wish to move from an “ailing body” to a “capable body” (e.g., “I feel this body is mine again”). The daily, measurable small victories—completing extra laps, improving a gateball shot—satisfy the desire to feel competent and in control. This aligns with desire-satisfaction theory because it is the “fulfillment of the participant’s own desire”for physical mastery, not an externally imposed standard, that generates well-being.

Theme 2 (Routinized communitas) addresses the desire for temporal structure and emotional connection. Retirement abruptly removes the social clock, leaving a “narrative vacuum” and unstructured time. Participants’ desire for routine, predictability, and shared emotional experience is satisfied through regular group activities (e.g., “happy shift”). The production of collective effervescence—however routinized—directly fulfills the desire to belong and to feel emotionally energized in the company of others.

Theme 3 (Role transformation and social capital) addresses the desire for social value and recognition. Older adults desire to feel useful, needed, and respected. Sports communities provide new roles (e.g., “Captain Chen,” “chief logistics officer”) that are earned through present contributions rather than past status. The satisfaction of this desire is evident in statements such as “I feel useful, like an indispensable “old ox” in this group.” The accumulation of weak-tie social capital further satisfies the desire for practical and emotional support outside family circles.

Theme 4 (Narrative identity shift) addresses the desire for a coherent, positive self-story. Participants rejected the culturally dominant decline narrative and actively re-authored their identities as growing, striving, and valuable persons (e.g., “second spring”). Sports participation provides raw narrative materials—achievements, recognitions, challenges—that enable the satisfaction of the desire to see one’s life as meaningful and progressive rather than as a mere waiting period.

Crucially, desire-satisfaction theory does not assume that all desires are equally achievable or beneficial. In the context of later life, the theory’s focus on *informed* desires—those based on realistic self-knowledge and feasible opportunities—is particularly relevant. The older adults in this study did not pursue unattainable youth; they recalibrated their desires toward achievable, meaningful goals within their physical and social capacities. Sports participation served as the practical arena where this recalibration and subsequent satisfaction took place. Thus, the meaning-centered model can be understood as an empirically grounded specification of how desire-satisfaction operates in the specific domain of later-life sports participation. This integration strengthens the dialogue between the study’s findings and broader well-being literature, showing that the pursuit and fulfillment of context-sensitive desires is a key mechanism linking activity to well-being in aging populations.

### 5.4 Limitations and future research

Several limitations of this study should be acknowledged. First, the sample was drawn from urban areas in northeastern China, and all participants were involved in organized sports activities. Findings may not directly transfer to rural older adults or those who engage in solitary, unorganized physical activities (e.g., individual walking at home). Future research should explore whether and how the meaning-centered model applies across different geographic, cultural, and activity contexts.

Second, the cross-sectional design captures well-being experiences at a single point in time, albeit with retrospective narratives. A longitudinal design could better capture the temporal unfolding of self-reconstruction processes, for example, how routinized communitas accumulates emotional energy over months or years.

Third, while this study intentionally excluded solitary activities to focus on socially rich contexts, many older adults do exercise alone. Their pathways to meaning-making may differ—perhaps through different mechanisms such as mindfulness, contemplation, or connection to nature. Comparative studies between organized and solitary sports participation would be valuable.

Fourth, the author’s identity as a male, non-retired researcher may have shaped participants’ willingness to disclose certain experiences. Although prolonged engagement and member checking were used to mitigate bias, future studies could include older peer researchers or co-researchers to reduce age-related power imbalances.

Finally, this study focused on well-being as the outcome. Future research could examine negative experiences—such as exclusion, competition, or injury within sports groups—and how older adults navigate these challenges while preserving or reconstructing meaning.

## 6 Conclusion

### 6.1 Summary of theoretical contributions

This qualitative study, grounded in constructivist grounded theory and phenomenological inquiry with 24 older adults, has revealed that the well-being enhancement associated with sports participation is not a simple linear “stimulus–response” relationship but a multidimensional meaning-making process centered on self-reconstruction. Across bodily, socio-temporal, relational, and narrative dimensions, older adults actively transform their experience of aging—from loss to agency, from isolation to belonging, from decline to growth. This study proposes a “meaning-centered” theoretical model that integrates these dimensions into a coherent logic chain: pursuing meaning → self-reconstruction → well-being experience.

In dialogue with existing theories, this study makes three specific contributions. First, it extends interaction ritual chain theory by identifying routinized communitas—a daily, low-intensity, high-frequency form of collective effervescence that regularly produces emotional energy for older adults. Second, it re-theorizes the mediating mechanism from static psychological resilience to dynamic narrative meaning-making, showing how sports participation provides narrative materials for re-authoring a progressive aging identity. Third, it complements active aging frameworks by foregrounding older adults as meaning creators rather than merely service recipients, arguing that meaningful aging is as important as healthy or productive aging.

### 6.2 Practical implications

The findings carry concrete implications for policy makers, community practitioners, and cultural discourses.

For policy makers: Senior sports support should shift from a management mindset to an ecological enabling mindset. Instead of standardizing top-down programs, policies should provide flexible, low-barrier resources that support older adults’ spontaneous meaning-making practices. Examples include: small-scale community sports grants, simplified venue access procedures, and the recognition and training of local sports leaders (e.g., square dance captains, gateball organizers). The goal is to enable, not replace, older adults’ agency.

For community practitioners: Social workers and activity organizers should reframe their role from “program deliverers” to platform builders and relationship facilitators. Creating safe, friendly, inclusive physical and social environments—where different sports groups can emerge organically—is more important than prescribing specific activities. Practitioners can also actively identify and support emerging community leaders, who play a crucial role in sustaining routinized communitas.

For cultural and media discourses: Public narratives about senior sports should move beyond health instrumentalism (“exercise prevents falls”) to recognize their deeper psychosocial value: spiritual comfort, social connection, and meaning confirmation. Media stories that celebrate older adults’ agency, creativity, and mutual support—rather than portraying them as frail and dependent—can help reduce ageism and foster an “age-friendly” culture where later life is seen as a period of continued growth and value.

### 6.3 Concluding remarks

The older adults in this study are not passive recipients of aging or policy. They are active meaning-makers, daily engaged in a quiet but profound project of self-reconstruction through the seemingly ordinary acts of dancing, walking, cycling, and practicing Tai Chi together. Their stories remind us that well-being in later life is not something that can be delivered or prescribed from above; it is something that is practiced, shared, and narrated from within. By recognizing, respecting, and enabling these spontaneous, life-affirming practices, we can move closer to a society where aging is not feared but celebrated—where every older person can say, with genuine conviction, “I can, I belong, I matter.”

## Data Availability

The datasets presented in this study can be found in online repositories. The names of the repository/repositories and accession number(s) can be found in the article/supplementary material.
